# *N* ^6^,3′-*cyclo*-5′-*O*-Cyano­methyl­thymidine

**DOI:** 10.1107/S1600536810019379

**Published:** 2010-05-29

**Authors:** Jingbo Sun, Kun Yang, Ronghui Duan, Jinchang Wu

**Affiliations:** a College of Chemistry, Jilin University, Changchun 130012, People’s Republic of China

## Abstract

The title compound, C_19_H_20_N_4_O_4_, is a cyclo­nucleoside with a C—N linkage. The furan­ose ring adopts a twist C3′-*endo*/C2′-*exo* (close to ^3^ *T*_2_) conformation with a pseudorotational phase angle (*P*) of 8.1° and puckering amplitude (*v*_m_) of 30.6°. The orientation of the pyrimidine ring with respect to the sugar group is *anti*. One intra­molecular C—H⋯O hydrogen bond is observed. The packing features an N—H⋯O hydrogen bond.

## Related literature

For nucleosides, see: Kaur *et al.* (2007 ▶); Imanishi & Satoshi (2002 ▶); Len *et al.* (2008 ▶); Mieczkowski *et al.* (2010 ▶); Sanger (1984 ▶); Altona & Sundaralingam (1972 ▶, 1973 ▶); Zhou & Chattopadhyaya (2009 ▶).
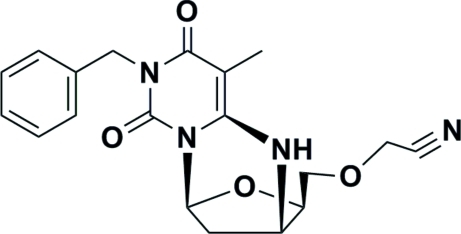


## Experimental

### 

#### Crystal data

C_19_H_20_N_4_O_4_
*M* *_r_* = 368.39
Orthorhombic, 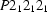

*a* = 10.1682 (7) Å
*b* = 11.0867 (8) Å
*c* = 15.8882 (11) Å
*V* = 1791.1 (2) Å^3^
*Z* = 4
Mo *K*α radiation
μ = 0.10 mm^−1^
*T* = 295 K
0.15 × 0.11 × 0.09 mm

#### Data collection

Bruker SMART 1000 diffractometer
Absorption correction: multi-scan (*SADABS*; Bruker, 2001 ▶) *T*_min_ = 0.985, *T*_max_ = 0.991
10105 measured reflections
2036 independent reflections
1756 reflections with *I* > 2σ(*I*)
*R*_int_ = 0.031

#### Refinement

*R*[*F* ^2^ > 2σ(*F* ^2^)] = 0.034
*wR*(*F* ^2^) = 0.079
*S* = 1.04
2036 reflections
249 parameters
H atoms treated by a mixture of independent and constrained refinement
Δρ_max_ = 0.18 e Å^−3^
Δρ_min_ = −0.15 e Å^−3^

### 

Data collection: *SMART* (Bruker, 1998 ▶); cell refinement: *SAINT* (Bruker, 1998 ▶); data reduction: *SAINT*; program(s) used to solve structure: *SHELXS97* (Sheldrick, 2008 ▶); program(s) used to refine structure: *SHELXL97* (Sheldrick, 2008 ▶); molecular graphics: *SHELXTL* (Sheldrick, 2008 ▶); software used to prepare material for publication: *SHELXL97* (Sheldrick, 2008 ▶).

## Supplementary Material

Crystal structure: contains datablocks global, I. DOI: 10.1107/S1600536810019379/om2332sup1.cif

Structure factors: contains datablocks I. DOI: 10.1107/S1600536810019379/om2332Isup2.hkl

Additional supplementary materials:  crystallographic information; 3D view; checkCIF report

## Figures and Tables

**Table 1:** Hydrogen-bond geometry (Å, °)

*D*—H⋯*A*	*D*—H	H⋯*A*	*D*⋯*A*	*D*—H⋯*A*
N1—H1⋯O3^i^	0.85 (2)	2.12 (2)	2.938 (2)	161 (2)
C6—H6⋯O2	0.93	2.89	3.492 (3)	123

Symmetry code: (i) 
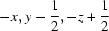
.

**Fig. 1.**
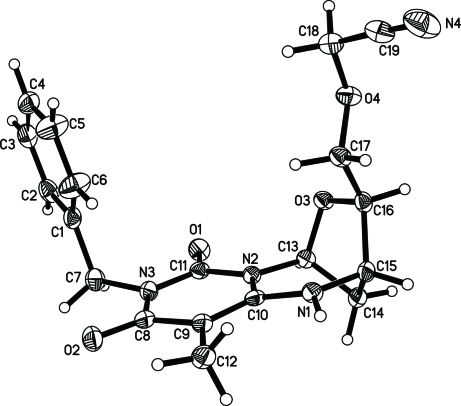
View of the molecule of the title compound showing the atom labelling scheme. Displacement ellipsoids are drawn at the 30% probability level.

## supplementary crystallographic information

### Comment

Nucleosides with restricted conformations have attracted attention with the development of LNAs (Kaur, *et al.* 2007) and BNAs (Imanishi & Satoshi, 2002) in recent years. Cyclonucleosides in which there is a linkage between nucleobase and the sugar moiety of nucleosides constrains both the puckering of the sugar ring and the glycosyl torsion angle (Len, *et al.*, 2008). Although many cyclonucleosides were synthesized since the early years (Mieczkowski, *et al.*, 2010), the study of their conformations and evaluation of their biological properties is relatively rare. Here we report the structure of a cyclonucleoside with a C—N linkage between C6 and C2' of thymidine.

In the title compound (Fig. 1), the five-membered ribose ring C13/C14/C15/C16/O3 adopts a North conformation (close to ^3^T_2_), with a pseudorotational phase angle (*P*) of 8.1° and puckering amplitude (*v*_m_) of 30.6° (Sanger, 1984; Altona & Sundaralingam, 1972; Altona & Sundaralingam, 1973). The glycosydic torsion angle ξ (O3—C6—N1—C1) of 99.4 (18)° shows the orientation of the pyrimidine ring to be anti with respect to the sugar group. The torsion angle ρ (C8—C9—C12—N3) is 174.15 (15)°. The C15—N1 and C10—N1 bond lengths in the linkage are 1.463 (2) and 1.355 (2)Å, it is clearly that the bond between the nucleobase and linkage is nearly double bond because of conjugation. The linkage bond angle of C10—N1—C15 is 121.36 (16)°.

### Experimental

The compound was separated from refluxing toluene solution of 1-(3-azido-2,3-dideoxy-5-cyanomethyl-5-deoxy-β-*D*-threo-pentofuranosy1) thymine (manuscript in preparation). It was crystallized slowly from a mixture of ethanol and ethyl acetate(1:2) at 298 K.

### Refinement

The C-bound H atoms were positioned geometrically with C—H = 0.93 (aromatic carbon), 0.97 (methylene), 0.96 (methyl) and 0.98 (methenyl)Å, and allowed to ride on their parent atoms in the riding model approximation with *U*_iso_(H) = 1.2 (1.5 for methyl) *U*_eq_(C). The atom H1 was located in a difference Fourier map and refined isotropically. In the absence of significant anomalous scattering effect, Friedel opposites were merged.

### Crystal data

**Table:**

C_19_H_20_N_4_O_4_	*F*(000) = 776
*M**_r_* = 368.39	*D*_x_ = 1.366 Mg m^−^^3^
Orthorhombic, *P*2_1_2_1_2_1_	Mo *K*α radiation, λ = 0.71073 Å
Hall symbol: P 2ac 2ab	Cell parameters from 2856 reflections
*a* = 10.1682 (7) Å	θ = 2.2–26.1°
*b* = 11.0867 (8) Å	µ = 0.10 mm^−^^1^
*c* = 15.8882 (11) Å	*T* = 295 K
*V* = 1791.1 (2) Å^3^	Block, colourless
*Z* = 4	0.15 × 0.11 × 0.09 mm

### Data collection

**Table:**

Bruker SMART 1000 diffractometer	2036 independent reflections
Radiation source: fine-focus sealed tube	1756 reflections with *I* > 2σ(*I*)
graphite	*R*_int_ = 0.031
Detector resolution: 9.00 pixels mm^-1^	θ_max_ = 26.1°, θ_min_ = 2.2°
φ and ω scans	*h* = −12→10
Absorption correction: multi-scan (*SADABS*; Bruker, 2001)	*k* = −13→13
*T*_min_ = 0.985, *T*_max_ = 0.991	*l* = −19→18
10105 measured reflections	

### Refinement

**Table:**

Refinement on *F*^2^	Primary atom site location: structure-invariant direct methods
Least-squares matrix: full	Secondary atom site location: difference Fourier map
*R*[*F*^2^ > 2σ(*F*^2^)] = 0.034	Hydrogen site location: inferred from neighbouring sites
*wR*(*F*^2^) = 0.079	H atoms treated by a mixture of independent and constrained refinement
*S* = 1.04	*w* = 1/[σ^2^(*F*_o_^2^) + (0.0361*P*)^2^ + 0.3497*P*] where *P* = (*F*_o_^2^ + 2*F*_c_^2^)/3
2036 reflections	(Δ/σ)_max_ < 0.001
249 parameters	Δρ_max_ = 0.18 e Å^−^^3^
0 restraints	Δρ_min_ = −0.15 e Å^−^^3^

### Special details

**Table:**

Experimental. (See detailed section in the paper)
Geometry. All esds (except the esd in the dihedral angle between two l.s. planes) are estimated using the full covariance matrix. The cell esds are taken into account individually in the estimation of esds in distances, angles and torsion angles; correlations between esds in cell parameters are only used when they are defined by crystal symmetry. An approximate (isotropic) treatment of cell esds is used for estimating esds involving l.s. planes.
Refinement. Refinement of *F*^2^ against ALL reflections. The weighted *R*-factor wR and goodness of fit *S* are based on *F*^2^, conventional *R*-factors *R* are based on *F*, with *F* set to zero for negative *F*^2^. The threshold expression of *F*^2^ > σ(*F*^2^) is used only for calculating *R*-factors(gt) etc. and is not relevant to the choice of reflections for refinement. *R*-factors based on *F*^2^ are statistically about twice as large as those based on *F*, and *R*- factors based on ALL data will be even larger.

### Fractional atomic coordinates and isotropic or equivalent isotropic displacement parameters (Å^2^)

**Table:**

	*x*	*y*	*z*	*U*_iso_*/*U*_eq_	
O1	0.26037 (18)	0.41221 (14)	0.18977 (11)	0.0419 (4)	
O2	0.2659 (2)	0.16696 (16)	−0.04016 (11)	0.0476 (5)	
O3	0.01006 (15)	0.29016 (14)	0.30360 (10)	0.0331 (4)	
O4	−0.26649 (17)	0.29459 (15)	0.27577 (11)	0.0414 (4)	
N1	0.0436 (2)	0.04989 (18)	0.20934 (13)	0.0322 (5)	
H1	0.034 (2)	−0.023 (2)	0.1935 (14)	0.027 (6)*	
N2	0.16403 (18)	0.22763 (16)	0.20033 (12)	0.0280 (4)	
N3	0.2616 (2)	0.29017 (17)	0.07452 (12)	0.0345 (5)	
N4	−0.5475 (2)	0.1380 (3)	0.2508 (2)	0.0720 (8)	
C1	0.2157 (3)	0.4766 (2)	−0.00647 (16)	0.0382 (6)	
C2	0.2429 (3)	0.5985 (2)	−0.01234 (16)	0.0434 (7)	
H2	0.3247	0.6274	0.0044	0.052*	
C3	0.1488 (3)	0.6782 (2)	−0.04317 (17)	0.0481 (7)	
H3	0.1688	0.7598	−0.0476	0.058*	
C4	0.0270 (3)	0.6379 (3)	−0.06705 (17)	0.0493 (7)	
H4	−0.0360	0.6917	−0.0867	0.059*	
C5	−0.0002 (3)	0.5186 (3)	−0.0617 (2)	0.0651 (9)	
H5	−0.0823	0.4903	−0.0784	0.078*	
C6	0.0925 (3)	0.4386 (3)	−0.0316 (2)	0.0612 (9)	
H6	0.0715	0.3571	−0.0282	0.073*	
C7	0.3192 (3)	0.3888 (2)	0.02538 (17)	0.0415 (6)	
H7A	0.3665	0.3554	−0.0222	0.050*	
H7B	0.3819	0.4321	0.0601	0.050*	
C8	0.2318 (3)	0.1800 (2)	0.03381 (15)	0.0343 (5)	
C9	0.1612 (2)	0.0937 (2)	0.08223 (15)	0.0316 (5)	
C10	0.1238 (2)	0.12081 (19)	0.16251 (15)	0.0277 (5)	
C11	0.2314 (2)	0.3165 (2)	0.15690 (15)	0.0314 (5)	
C12	0.1264 (3)	−0.0252 (2)	0.04249 (17)	0.0399 (6)	
H12A	0.1788	−0.0880	0.0672	0.060*	
H12B	0.1433	−0.0215	−0.0169	0.060*	
H12C	0.0349	−0.0420	0.0518	0.060*	
C13	0.1405 (2)	0.2471 (2)	0.29099 (15)	0.0301 (5)	
H13	0.2054	0.3027	0.3149	0.036*	
C14	0.1430 (2)	0.1270 (2)	0.33571 (15)	0.0348 (6)	
H14A	0.1374	0.1356	0.3964	0.042*	
H14B	0.2197	0.0794	0.3210	0.042*	
C15	0.0176 (2)	0.0765 (2)	0.29797 (14)	0.0317 (5)	
H15	−0.0133	0.0051	0.3285	0.038*	
C16	−0.0762 (2)	0.1842 (2)	0.30890 (15)	0.0315 (5)	
H16	−0.1137	0.1809	0.3657	0.038*	
C17	−0.1865 (2)	0.1961 (2)	0.24724 (16)	0.0350 (5)	
H17A	−0.1524	0.2125	0.1914	0.042*	
H17B	−0.2376	0.1223	0.2452	0.042*	
C18	−0.3794 (3)	0.3126 (3)	0.22543 (18)	0.0475 (7)	
H18A	−0.3540	0.3150	0.1666	0.057*	
H18B	−0.4192	0.3895	0.2395	0.057*	
C19	−0.4758 (3)	0.2153 (3)	0.23850 (19)	0.0499 (7)	

### Atomic displacement parameters (Å^2^)

**Table:**

	*U*^11^	*U*^22^	*U*^33^	*U*^12^	*U*^13^	*U*^23^
O1	0.0471 (11)	0.0284 (9)	0.0502 (10)	−0.0092 (9)	0.0064 (9)	−0.0036 (8)
O2	0.0649 (13)	0.0443 (11)	0.0336 (10)	0.0056 (10)	0.0107 (9)	0.0019 (8)
O3	0.0327 (9)	0.0251 (8)	0.0416 (9)	0.0000 (7)	0.0067 (8)	−0.0025 (7)
O4	0.0349 (9)	0.0366 (9)	0.0525 (11)	0.0061 (8)	0.0015 (8)	−0.0049 (8)
N1	0.0373 (12)	0.0202 (10)	0.0391 (12)	−0.0018 (9)	0.0062 (9)	−0.0028 (9)
N2	0.0299 (10)	0.0229 (9)	0.0313 (10)	−0.0010 (8)	0.0031 (8)	−0.0003 (8)
N3	0.0366 (11)	0.0300 (10)	0.0368 (11)	−0.0005 (9)	0.0073 (9)	0.0067 (9)
N4	0.0401 (15)	0.0722 (18)	0.104 (2)	−0.0030 (14)	0.0069 (16)	−0.0095 (18)
C1	0.0470 (16)	0.0323 (13)	0.0354 (13)	−0.0036 (11)	0.0094 (12)	0.0060 (10)
C2	0.0494 (16)	0.0363 (14)	0.0445 (15)	−0.0087 (14)	0.0147 (13)	−0.0039 (11)
C3	0.075 (2)	0.0226 (12)	0.0470 (16)	0.0023 (13)	0.0218 (15)	0.0018 (12)
C4	0.063 (2)	0.0403 (16)	0.0445 (16)	0.0120 (14)	0.0054 (14)	0.0070 (12)
C5	0.0517 (18)	0.0491 (19)	0.094 (3)	−0.0004 (15)	−0.0155 (19)	0.0119 (17)
C6	0.0521 (19)	0.0343 (15)	0.097 (3)	−0.0099 (13)	−0.0142 (18)	0.0167 (16)
C7	0.0393 (14)	0.0401 (15)	0.0451 (16)	−0.0067 (12)	0.0080 (12)	0.0072 (12)
C8	0.0381 (13)	0.0304 (12)	0.0345 (13)	0.0092 (11)	0.0015 (11)	0.0026 (10)
C9	0.0340 (13)	0.0279 (12)	0.0330 (13)	0.0035 (10)	0.0009 (10)	−0.0009 (10)
C10	0.0262 (11)	0.0210 (11)	0.0359 (13)	0.0043 (9)	−0.0012 (10)	0.0011 (9)
C11	0.0281 (12)	0.0247 (11)	0.0416 (13)	0.0001 (9)	0.0025 (11)	0.0023 (10)
C12	0.0511 (16)	0.0314 (13)	0.0373 (14)	0.0026 (12)	−0.0013 (13)	−0.0036 (11)
C13	0.0291 (12)	0.0286 (12)	0.0327 (12)	−0.0011 (9)	0.0008 (10)	−0.0021 (10)
C14	0.0342 (14)	0.0377 (13)	0.0325 (13)	0.0012 (11)	−0.0003 (11)	0.0055 (11)
C15	0.0353 (13)	0.0267 (12)	0.0329 (13)	−0.0023 (10)	0.0058 (11)	0.0048 (10)
C16	0.0342 (12)	0.0276 (12)	0.0326 (12)	−0.0039 (10)	0.0080 (10)	0.0007 (10)
C17	0.0332 (13)	0.0281 (12)	0.0437 (14)	−0.0007 (10)	0.0063 (11)	−0.0036 (11)
C18	0.0406 (15)	0.0482 (16)	0.0539 (17)	0.0105 (13)	0.0022 (13)	0.0076 (13)
C19	0.0331 (14)	0.0575 (18)	0.0592 (18)	0.0073 (14)	0.0008 (13)	−0.0053 (15)

### Geometric parameters (Å, °)

**Table:**

O1—C11	1.219 (3)	C5—C6	1.380 (4)
O2—C8	1.234 (3)	C5—H5	0.9300
O3—C13	1.424 (3)	C6—H6	0.9300
O3—C16	1.468 (3)	C7—H7A	0.9700
O4—C18	1.413 (3)	C7—H7B	0.9700
O4—C17	1.435 (3)	C8—C9	1.422 (3)
N1—C10	1.355 (3)	C9—C10	1.365 (3)
N1—C15	1.463 (3)	C9—C12	1.504 (3)
N1—H1	0.85 (2)	C12—H12A	0.9600
N2—C11	1.384 (3)	C12—H12B	0.9600
N2—C10	1.390 (3)	C12—H12C	0.9600
N2—C13	1.476 (3)	C13—C14	1.509 (3)
N3—C11	1.376 (3)	C13—H13	0.9800
N3—C8	1.415 (3)	C14—C15	1.517 (3)
N3—C7	1.466 (3)	C14—H14A	0.9700
N4—C19	1.142 (4)	C14—H14B	0.9700
C1—C6	1.381 (4)	C15—C16	1.539 (3)
C1—C2	1.382 (4)	C15—H15	0.9800
C1—C7	1.520 (4)	C16—C17	1.495 (3)
C2—C3	1.392 (4)	C16—H16	0.9800
C2—H2	0.9300	C17—H17A	0.9700
C3—C4	1.370 (4)	C17—H17B	0.9700
C3—H3	0.9300	C18—C19	1.472 (4)
C4—C5	1.354 (4)	C18—H18A	0.9700
C4—H4	0.9300	C18—H18B	0.9700
			
C13—O3—C16	107.23 (16)	O1—C11—N2	121.7 (2)
C18—O4—C17	112.91 (19)	N3—C11—N2	115.7 (2)
C10—N1—C15	121.4 (2)	C9—C12—H12A	109.5
C10—N1—H1	117.2 (16)	C9—C12—H12B	109.5
C15—N1—H1	117.0 (16)	H12A—C12—H12B	109.5
C11—N2—C10	122.47 (19)	C9—C12—H12C	109.5
C11—N2—C13	117.56 (19)	H12A—C12—H12C	109.5
C10—N2—C13	119.92 (19)	H12B—C12—H12C	109.5
C11—N3—C8	124.78 (19)	O3—C13—N2	109.69 (18)
C11—N3—C7	115.9 (2)	O3—C13—C14	104.21 (18)
C8—N3—C7	119.09 (19)	N2—C13—C14	109.15 (19)
C6—C1—C2	117.4 (3)	O3—C13—H13	111.2
C6—C1—C7	121.9 (2)	N2—C13—H13	111.2
C2—C1—C7	120.6 (2)	C14—C13—H13	111.2
C1—C2—C3	120.4 (3)	C13—C14—C15	97.21 (19)
C1—C2—H2	119.8	C13—C14—H14A	112.3
C3—C2—H2	119.8	C15—C14—H14A	112.3
C4—C3—C2	120.8 (3)	C13—C14—H14B	112.3
C4—C3—H3	119.6	C15—C14—H14B	112.3
C2—C3—H3	119.6	H14A—C14—H14B	109.9
C5—C4—C3	119.1 (3)	N1—C15—C14	107.63 (19)
C5—C4—H4	120.5	N1—C15—C16	112.16 (19)
C3—C4—H4	120.5	C14—C15—C16	100.96 (18)
C4—C5—C6	120.6 (3)	N1—C15—H15	111.8
C4—C5—H5	119.7	C14—C15—H15	111.8
C6—C5—H5	119.7	C16—C15—H15	111.8
C5—C6—C1	121.6 (3)	O3—C16—C17	109.88 (18)
C5—C6—H6	119.2	O3—C16—C15	104.13 (17)
C1—C6—H6	119.2	C17—C16—C15	117.36 (19)
N3—C7—C1	112.2 (2)	O3—C16—H16	108.4
N3—C7—H7A	109.2	C17—C16—H16	108.4
C1—C7—H7A	109.2	C15—C16—H16	108.4
N3—C7—H7B	109.2	O4—C17—C16	106.58 (19)
C1—C7—H7B	109.2	O4—C17—H17A	110.4
H7A—C7—H7B	107.9	C16—C17—H17A	110.4
O2—C8—N3	118.5 (2)	O4—C17—H17B	110.4
O2—C8—C9	125.3 (2)	C16—C17—H17B	110.4
N3—C8—C9	116.2 (2)	H17A—C17—H17B	108.6
C10—C9—C8	119.9 (2)	O4—C18—C19	110.9 (2)
C10—C9—C12	121.3 (2)	O4—C18—H18A	109.5
C8—C9—C12	118.8 (2)	C19—C18—H18A	109.5
N1—C10—C9	123.6 (2)	O4—C18—H18B	109.5
N1—C10—N2	115.7 (2)	C19—C18—H18B	109.5
C9—C10—N2	120.6 (2)	H18A—C18—H18B	108.0
O1—C11—N3	122.6 (2)	N4—C19—C18	177.4 (3)
			
C6—C1—C2—C3	−0.5 (4)	C8—N3—C11—O1	179.1 (2)
C7—C1—C2—C3	178.7 (2)	C7—N3—C11—O1	−6.0 (3)
C1—C2—C3—C4	1.0 (4)	C8—N3—C11—N2	−1.7 (3)
C2—C3—C4—C5	−1.1 (4)	C7—N3—C11—N2	173.2 (2)
C3—C4—C5—C6	0.8 (5)	C10—N2—C11—O1	176.3 (2)
C4—C5—C6—C1	−0.3 (6)	C13—N2—C11—O1	−6.4 (3)
C2—C1—C6—C5	0.2 (5)	C10—N2—C11—N3	−2.9 (3)
C7—C1—C6—C5	−179.0 (3)	C13—N2—C11—N3	174.4 (2)
C11—N3—C7—C1	−80.4 (3)	C16—O3—C13—N2	85.9 (2)
C8—N3—C7—C1	94.8 (3)	C16—O3—C13—C14	−30.8 (2)
C6—C1—C7—N3	−35.9 (4)	C11—N2—C13—O3	99.4 (2)
C2—C1—C7—N3	145.0 (2)	C10—N2—C13—O3	−83.3 (2)
C11—N3—C8—O2	−179.0 (2)	C11—N2—C13—C14	−147.0 (2)
C7—N3—C8—O2	6.3 (3)	C10—N2—C13—C14	30.3 (3)
C11—N3—C8—C9	2.4 (3)	O3—C13—C14—C15	48.7 (2)
C7—N3—C8—C9	−172.4 (2)	N2—C13—C14—C15	−68.4 (2)
O2—C8—C9—C10	−177.0 (2)	C10—N1—C15—C14	−35.7 (3)
N3—C8—C9—C10	1.5 (3)	C10—N1—C15—C16	74.5 (3)
O2—C8—C9—C12	1.4 (4)	C13—C14—C15—N1	70.8 (2)
N3—C8—C9—C12	179.9 (2)	C13—C14—C15—C16	−46.9 (2)
C15—N1—C10—C9	173.5 (2)	C13—O3—C16—C17	−126.6 (2)
C15—N1—C10—N2	−8.5 (3)	C13—O3—C16—C15	−0.1 (2)
C8—C9—C10—N1	172.0 (2)	N1—C15—C16—O3	−84.0 (2)
C12—C9—C10—N1	−6.3 (4)	C14—C15—C16—O3	30.3 (2)
C8—C9—C10—N2	−5.9 (3)	N1—C15—C16—C17	37.7 (3)
C12—C9—C10—N2	175.7 (2)	C14—C15—C16—C17	152.0 (2)
C11—N2—C10—N1	−171.3 (2)	C18—O4—C17—C16	−176.96 (19)
C13—N2—C10—N1	11.5 (3)	O3—C16—C17—O4	−67.2 (2)
C11—N2—C10—C9	6.8 (3)	C15—C16—C17—O4	174.11 (18)
C13—N2—C10—C9	−170.4 (2)	C17—O4—C18—C19	71.4 (3)

### Hydrogen-bond geometry (Å, °)

**Table:**

*D*—H···*A*	*D*—H	H···*A*	*D*···*A*	*D*—H···*A*
N1—H1···O3^i^	0.85 (2)	2.12 (2)	2.938 (2)	161 (2)
C6—H6···O2	0.93	2.89	3.492 (3)	123

Symmetry codes: (i) −*x*, *y*−1/2, −*z*+1/2.
